# Inflammatory Bowel Disease (IBD)—A Textbook Case for Multi-Centric Banking of Human Biological Materials

**DOI:** 10.3389/fmed.2019.00230

**Published:** 2019-10-18

**Authors:** Isabelle Cleynen, Loes Linsen, Sare Verstockt, Bram Verstockt, Vera Ballet, Eline Vandeput, Gert Van Assche, Marc Ferrante, Kristel Van Landuyt, Séverine Vermeire, Nadine Ectors

**Affiliations:** ^1^Laboratory for Complex Genetics, Department of Human Genetics, KU Leuven, Leuven, Belgium; ^2^Activity Center Biobanking, University Hospitals Leuven, Leuven, Belgium; ^3^Department of Chronic Diseases, Metabolism and Ageing (CHROMETA), Translational Research Center for Gastrointestinal Disorders (TARGID), KU Leuven, Leuven, Belgium; ^4^Department of Gastroenterology and Hepatology, University Hospitals Leuven, KU Leuven, Leuven, Belgium

**Keywords:** inflammatory bowel disease, biobank, BBMRI.be, PSI, BBMRI.nl, Crohn's disease, ulcerative colitis

## Abstract

Inflammatory bowel disease (IBD) is a chronic relapsing inflammatory condition affecting mainly the gastro-intestinal tract with two main entities: Crohn's disease (CD) and ulcerative colitis (UC). Although the exact mechanisms underlying the initial development of IBD are not fully understood, it is believed that an abnormal immune response is elicited against the intestinal microbiota in genetically predisposed individuals. Crucial elements of the etiopathogenesis have been elucidated by research using human biological materials. The estimated prevalence of IBD is 0.5% in the Western world. Although incidence rates are increasing, both conditions are not “common” in general terms mandating a multicentric approach. Biological material from numerous Belgian patients have been collected over time in a number of university hospitals in Belgium (UH Ghent: 800 CD patients, 350 UC patients, 600 normal controls; UH Leuven: 2,600 CD patients, 1,380 UC patients, 98 IC/IBDU patients, 6,260 normal controls). Within the setting of the Flemish Center Medical Innovation (CMI) initiative and later on the Flemish biobank network a prospective study was set-up across three Belgian IBD centers (University Hospitals Brussels, Ghent, and Leuven). Human biological materials and data have been collected prospectively from newly diagnosed CD and UC patients. The analyses hereof have generated new insights which have been published in the most renowned journals. The approach of well-thought off, multi-centric, structured, and systematic biobanking has proven to be a success-story and thus a textbook case for multi-centric banking of human biological materials. This story is being told in this article.

## Introduction

A limited number of European biobanking initiatives relate to Inflammatory Bowel Diseases (IBD). These initiatives have shown to be highly effective in output, output as quantified by numbers of publications, grants obtained, multicentric collaborations. Unfortunately, they also confirmed the greatest fear and risk for biobanks i.e., concerning sustainability.

### Inflammatory Bowel Diseases in a Nutshell

Inflammatory bowel disease (IBD) is a chronic inflammatory condition mainly—but not solely—affecting the gastro-intestinal tract. The two main subtypes are Crohn's disease (CD) and ulcerative colitis (UC). These two main forms of IBD have both overlapping and distinct clinical pathological features. About 11.2 million people are affected with IBD as of 2015 (1). Incidence and prevalence are also increasing since the 1950s (2, 3). Each year it newly occurs in 1–20 per 100,000 people, and 5–500 per 100,000 individuals are affected (2, 3). The disease is more common in North America and Europe than other regions, although incidence is also increasing in previously considered low risk groups (3). Often it begins in people aged 15–30 years or among those over 60^1^. Males and females appear to be affected in equal proportions (2). For both conditions, the etiopathogenesis is multifactorial (environmental and genetic). Numerous environmental risk factors have been identified by means of epidemiological studies: smoking, appendectomy, infections, antibiotics, diet and lifestyle, … (4). The genetic background of IBD has been extensively studied, with the identification of circa 240 genetic loci associated with IBD thus far (5–7). More recently, the microbiome—the bacterial content—of the gut has been demonstrated to play a crucial role (8). The clinical presentation (symptoms, onset) as well as the course and outcome of the disease are variable. Treatment options are medical and surgical. Standard treatment depends on the extent of involvement and disease severity. The goal is to induce remission initially, followed by prevention of relapse as long as possible. IBD can be treated with a number of medications including biologics and more recently bacterial recolonization. For some, the disease has a mild course, while for others surgery is necessary. The current classification system based on symptoms does not always predict which path the patient will take. Genetic data also shows the same uncertainty. Researchers have calculated the genetic risk scores of 30,000 IBD patients based on 160 loci that determine the predisposition to IBD (9). They discovered that Crohn's disease in the small intestine and Crohn's disease in the colon differed genetically as much as Crohn's disease in the colon compared to ulcerative colitis.

## Biobanking

### Belgium—University Hospitals

The University hospitals of Leuven (UZ Leuven) have a tradition of clinical care and research in IBD spreading over several generations. At the forefront of the internal medicine—gastroenterology G. Vantrappen, P. Rutgeerts, and S. Vermeire have carried on the tradition. Obtaining and investigating human body materials (HBM) have naturally always been part of these processes.

The creation of the VLECC—biobank (VLAAMS ERFELIJKHEIDSONDERZOEK CROHN EN COLITIS ULCEROSA; Flemish inheritance study of Crohn's and Ulcerative colitis) in 1997 was a turning point. The main aim was to collect serum, DNA, and clinical characteristics of IBD patients. The biobank collected serial serum samples of patients with IBD and healthy controls that gave informed consent to participate in this study. From then onwards, collection of HBM and associated data has been performed prospectively in a structured manner, and now also includes tissue (biopsies) and fecal samples. The biobank started as a monocentric initiative. Numerous projects on different topics have arisen from hereon: genetic studies; investigations on treatment with biologics; the issue of anti-TNF antibodies; effectiveness; and safety of biologics, role of genetics on the response to biologics, investigations on environmental factors, and the role of bacteria in the bowel, research on serologic markers … to name only a few. These projects in turn generated numerous multicenter projects, and has led to many publications in high-ranked international journals (Lancet, Nature, Nature Genetics, Annals of Internal Medicine, Gastroenterology, Gut…) (9–14). The UZ KU Leuven Biobank operates its activities according to a quality management system, based on ISO 9001 for quality management systems, complemented with the biobank specific ISO 20387 standard and the ISBER Best Practices for biorepositories.

The Leuven biobank now contains DNA and serum of >4,000 IBD patients, >3,000 unaffected relatives, and 1,300 healthy controls, and a unique set of 60 multiple-affected IBD families. This large biobank with patient material has put the Leuven IBD group at the forefront of translational research in the field of IBD, not in the last in the IBD genetics field. Together with the IBD centers of Liège, Ghent, and Brussels, the Leuven IBD group conducted a Belgian genome-wide association study in 2007 (15), and co-founded the International IBD genetics consortium in the second half of the years 2000. They joined the combined analysis of GWAS across different countries (meta-analysis), leading to the identification of up to 99 confirmed loci in 2010 (16–18). In 2013, the largest international endeavor so far was initiated with the combined analysis of over 75,000 samples, including a few thousand samples from Belgium (Leuven, Ghent, Liège, and Brussels) (5) and culminating in the identification of over 200 loci associated with the risk to develop CD (19). Currently, the newest technologies are applied on these datasets (next-generation sequencing), and will undoubtedly lead to important new discoveries to further disentangle the genetic architecture of IBD and insights in disease pathogenesis.

### Belgium—Center Medical Innovation—CMI

The White Paper of FlandersBio in 2006, the VRW advice 120 regarding translational biomedical research in 2008, and the Technopolis business plan for the CMI (then the CTBI) in 2009, presented proposals for a translational biomedical research initiative in Flanders^2^ (20). The development of a strong center for translational biomedical research is also in accordance with the Flemish policy as an important part of the ViA Doorbraak “Flanders Innovation Centre,” the development of Flanders' Care, 2 and the policy plans of the Flemish Minister of Innovation, Ingrid Lieten^3^. The aim of the CMI was to ensure high quality translational biomedical research at an international level, based on cooperation between the Clinical Research Centers (CRCs) in Flanders and their partners, and the development and exploitation of the Flemish Biobank in this context. The growth of translational research in Flanders aims at contributing to better health care for the patient, and related to this, to economic and societal added value for the region and beyond.

The CMI has achieved important milestones in its preparatory task, in particular the preparation of the Flemish Biobank. Cooperation between the University Institutes and their CRCs is important for the prospective collection of biobank samples because the broadest possible population is covered in this way to collect as many samples as possible. On the basis of the inventory of the existing biobanks and the needs of translational biomedical research, “Focus Biobanks” were set up. High quality samples in the Focus Biobanks will be included in the Flemish Biobank with a central ICT backbone. The FFEU funds made it possible to establish the infrastructure for a good quality biobank in all the 4 affiliated CRCs, for CRC Leuven in cooperation with UHasselt.

**Figure 1:**
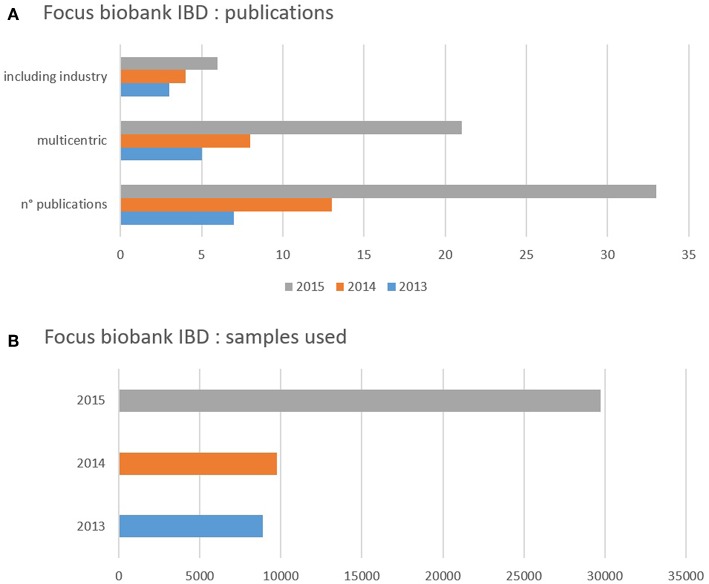
**(A,B)** Impact of biobanking initiative on samples used in research projects (in function of time) and initiating publication. **(A)** Shows the number of samples of human biological material used during 1 year (2013 blue, 2014 orange, 2015 gray). The figure demonstrates a clear increase in the use of samples over time which correlates with the activities in the biobank but also and more importantly with the output in publications **(B)**. **(B)** Shows the number of publications (*n*° publications) reporting on results achieved using the samples of human biological material during 1 year (2013 blue, 2014 orange, 2015 gray). The figure demonstrates an overall increase in number of publications. The total number of publications was split up highlighting two distinct categories: most of the studies were multicentric with an increasing number of the years. This highlights one of the needs/advantages in relatively rare diseases. The other highlight is the proportional increase of studies whereby the industry was one the scientific partners (participation not limited to sponsoring) which demonstrates one of the strengths of Biobanking.

For the CRC Leuven, the IBD initiative was an obvious choice as “Focus Biobank.” Intermediary analyses related to the performance of the focus biobank demonstrated clear-cut correlations with use of HBM and scientific output (Figures 1A,B).

At the time of the final evaluation, data (minimum data sets—MDS) from this collected HBM were uploaded on the central ICT backbone of the CMI. By the end of 2017, the data of 70,347 samples of IBDs were visible. These collections were obtained prospectively in the course of time in the context of multiple specific research projects. The uploaded cases fit with the participation in the initiated projects within the CMI (focus biobank/research platforms lead Leuven).

Within the CMI setting different projects were started, a retrospective project (University Hospitals Leuven, Ghent) led by Ghent and a prospective multicenter HBM collection (University Hospitals Leuven, Ghent, Brussels) [“BIB” (biobank IBD) project] led by Leuven.

In the meantime the initial CMI initiative has been ended. After an initial funding provided by the Flemish Government (8 mio € for the set-up of 4 CRCs and a centralized IT backbone) the CMI stopped to exist because of lack of funding at the end of 2017.

**Figure 2:**
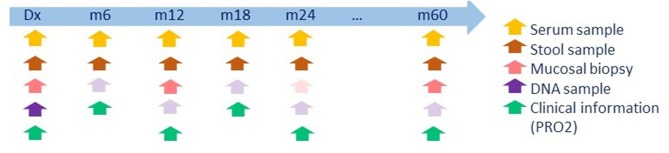
Time points and sample collection of the PANTHER cohort. Biopsies are only collected when a colonoscopy is required for clinical follow-up. If macroscopic inflammation is present, a biopsy is taken both at an inflamed site, and a macroscopically inactive site. Bright arrow colors indicate required samples; faint colors indicate optional samples. Dx, diagnosis; m, month.

However, the “BIB” (biobank IBD) project is still running and has been renamed to “PANTHER” (“Prognostic factors in patients with early Crohn's or colitis” study). The PANTHER study is aimed at characterizing newly diagnosed IBD patients and their disease progression. The design consists of a multicenter, standardized longitudinal follow-up, which not only includes phenomics, but also resampling at specified time points. End 2015, the IBD centers at the University Hospitals Leuven, Ghent and Brussels started to prospectively collect DNA, stool, serum, and endoscopy-derived intestinal biopsies (inflamed and uninflamed) of patients newly diagnosed with IBD (max. 6 months), naïve to biologic and immunosuppressive therapy, and no previous surgery related to the disease. Corticosteroid or 5-ASA use at diagnosis is noted in 8–9% of the cohort, and is considered as a covariate for downstream analyses. The included patients are followed longitudinally; clinical information (disease characteristics including standard biological measurements, medication use, demographics) is gathered; and different sample types are collected according to agreed SOPs and at predefined time points or when there is a marked change in disease characteristics or treatment policy (see Figure 2 for details). So far we included 234 patients (150 CD, 84 UC), with maximal follow-up at this moment of 3.6 years. Patient inclusion and follow-up has thus been ongoing since end 2015, a ratio of 80 inclusions per year. Inclusion rates and clinical characteristics are as expected, and is a continuous effort.

The previous years, the PANTHER cohort was predominantly used for diagnostic purposes, and thus to identify signatures that can separate patients from controls. Results from these analyses have been presented at (inter)national meetings and are being prepared for full paper submission. They are part of an ongoing PhD and master thesis project conducted at KU Leuven under the supervision of Prof. I. Cleynen and Prof. S. Vermeire. With continued inclusion and follow-up, the next stage of the project is to better understand disease heterogeneity to facilitate biomarker development and patient stratification. A new project proposal has been submitted to now identify biomarkers for disease progression by multi-dimensional holistic analyses of mucosal tissue and peripheral samples based on genetics (single cell), transcriptomics, and serology. With these unique datasets, we work toward a precision medicine approach aiming to tailor treatment of individual patients instead of a one-size-fits-all approach. The data gathered in this project will also allow data sharing with similar international consortia (see below Parelsnoer), and thus enable *ad hoc* international collaboration, data sharing, and access to larger sample sizes.

### The Netherlands—Parelsnoer Institute—PSI

The Parelsnoer Institute (PSI), established in 2007 by the Dutch Federation of University Medical Centers (NFU), offers researchers within the eight University Medical Centers and external researchers an infrastructure and standard procedures for the establishment, expansion and optimization of clinical biobanks for scientific research (21). By collecting and storing clinical data, images and human biomaterial together in a uniform manner from carefully documented patients suffering the same illness, large cohorts are established (the so-called “Pearls”) that enable broader scientific research.

To this aim, the prospective Dutch IBD Biobank was created. Gastroenterologists who specialized in treating patients with IBD in all eight Dutch university medical centers (UMC), together with a team of information architects and laboratory experts, built up the Dutch IBD Biobank. The main objective of the biobank is to facilitate the discovery of predictors (both epidemiological risk factors and biomarkers) for individual disease course and treatment response, by: providing full clinical records of patients describing their individual disease course over a prolonged period of time; providing high-quality biomaterials; standardizing patient data collection; and questionnaires during outpatient clinic visits and thereby improving clinical care (22).

In their article Spekhorst et al. refer to 3,388 patients with IBD enrolled in June 2014, IBD: 2,118 Crohn's disease (62.5%), 1,190 ulcerative colitis (35.1%), 74 IBD-unclassified (2.2%), and 6 IBD-indeterminate (0.2%) (22). Besides samples of HBM the Dutch IBD Biobank prospectively collects 225 standardized data items on various topics, including patient demographics, family history, diagnosis, disease activity, disease localization, results of physical examinations, radiographic imaging results, laboratory and endoscopy results, previous and current treatment, as well as a wide array of disease and treatment complications.

Similarly to the CMI project, after a large initial grant provided by the Dutch government to the Netherlands Federation of University Medical Centers facilitating the establishment of the Dutch IBD Biobank and seven similar biobanks ended in 2011, the Dutch UMCs had to fund the continuation of the Dutch IBD Biobank themselves, meaning a reduction of staff that assisted in patient inclusion in some centers. As a consequence, the enrolment of patients has slowed down in these centers (22). Here too the project kept on going.

### Europe—BBMRI—EU

The European Strategy Forum on Research Infrastructures (ESFRI) produced its first roadmap in October 2006 (23). Biobanking and BioMolecular resources Research Infrastructure (BBMRI) was one of the proposals, it is the largest infrastructure launched in Europe in health research. The ambitious mission of the BBMRI was to sustainably secure access to biological resources and data required for health-related research in Europe. The 7th European Union Framework program funded a 3-year BBMRI preparatory phase project (5 million Euros). Over time, a catalog from existing major population-based and clinical or disease-orientated biobanks was created with overall 20 million human biological samples (24). The members of BBMRI-ERIC were the European countries and intergovernmental organizations that have signed the BBMRI-ERIC Statutes. Founding Member States at that time were Austria, Belgium, Estonia, Finland, France, Germany, Greece, Italy, Malta, the Netherlands, and Sweden. BBMRI-ERIC primarily aims at establishing, operating, and developing a pan-European distributed research infrastructure of biobanks and biomolecular resources. This will facilitate the access to biological resources as well as biomedical facilities and support high-quality biomolecular and medical research. By nature it is a distributed infrastructure, in which biological samples and data are hosted by the European Member States biobanks.

**Figure 3:**
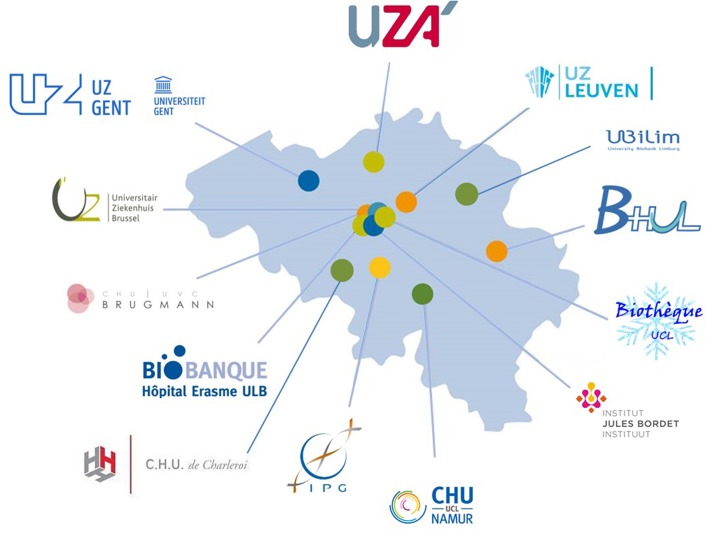
Overview of the Belgian biobanks currently connected to the BBMRI.be network. The BBMRI.be network connects 13 biobanks that are linked to public institutions such as hospitals, universities, and research centers.

BBMRI.be was set up in order to support the ever-increasing need of research with regard to quality control, access, transparency, and interconnectedness of biobanks. The scientific participation of Belgium in BBMRI-ERIC is exerted by a national node that was initiated by uniting the three existing Belgian network biobank initiatives i.e., Belgian Virtual Tumourbank project assigned to the Belgian Cancer Registry (BVT-BCR), Biothèque de la Fédération Wallonie-Bruxelles (BWB), and the Flemish Biobank Network (CMI). This network connects 13 biobanks that are linked to public institutions such as hospitals, universities and research centers and is included in the Directory of BBMRI-ERIC (Figure 3). BBMRI.be has matured into a solid partner network on biobanks in Belgium and has proven to reach out to a broader community beyond the founding partners. Data from the CMI focus biobank on IBD have been listed in the BBMRI-ERIC catalog as a clinical/disease-orientated biobank.

PSI and the Dutch IBD Biobank participate in the BBBMRI-ERIC project too as are part of the Biobanking and Biomolecular Resources Research Infrastructure of the Netherlands (BBMRI-NL). This is the Dutch national node of BBMRI-ERIC, the largest research infrastructure project in Europe (25). The BBMRI-NL biobank catalog is a searchable database, containing information on several Dutch bio- and databanks. To date, there are over 200 bio- and data collections listed.

Based on a data search in the BBBMRI-ERIC directory (performed on the 29th April 2019) 8 biobanks were found (as shown in Table 1) https://directory.bbmri-eric.eu/menu/main/app-molgenis-app-biobank-explorer/biobankexplorer?diagnosis_available=K50,K51 (26).

**Table 1:** Based on a data search in the BBBMRI-ERIC directory (performed on the 29th April 2019) 8 biobanks were found on https://directory.bbmri-eric.eu/menu/main/app-molgenis-app-biobank-explorer/biobankexplorer?diagnosis_available=K50,K51 (21).

	**Collection**	**Type**	**Materials**	**#Samples**
Nl	Academic medical center biobank			
	**Collection types:** Longitudinal, disease specific			
	**Juridical person:** AMC			
	Low countries Vedolizumab in Ulcerative Colitis study	Longitudinal	Serum, tissue (paraffin preserved)	100–1,000
	Low countries Vedolizumab in Crohn's disease study	Longitudinal	Serum, tissue (paraffin preserved)	100–1,000
	Predictive biomarkers and the role of the microbiome on treatment for inflammatory bowel disease	Disease specific, longitudinal	Feces, tissue (frozen), whole blood	1,000–10,000
B	Bimetra Biobank @ UZ Gent			
	**Collection types:** Longitudinal, disease specific, hospital			
	**Juridical person:** University Hospital Ghent			
	CRC Focus Collections @ Bimetra	Longitudinal, disease specific	DNA, plasma, serum, feces, other, whole blood, tissue (frozen)	19,848
	Inflammatory Bowel disease focus collection	Longitudinal, disease specific, hospital	Serum, plasma, DNA, RNA, tissue (frozen), feces, tissue (paraffin preserved)	2,617
Nl	BioBank Maastricht UMC			
	**Collection types:** Cohort, disease specific, longitudinal, population-based			
	**Juridical person:** Maastricht UMC+ (MUMC)			
	Inflammatory Bowel Disease Zuid Limburg Biobank	Cohort, disease specific, longitudinal, population-based	DNA, feces, other, plasma, RNA, serum, tissue (paraffin preserved)	1,000–10,000
B	Biobank-University Hospitals Leuven			
	**Collection types:** Disease specific			
	**Juridical person:** University Hospitals Leuven (B0383)			
	Inflammatory Bowel Disease (IBD)	Disease specific	DNA, plasma, serum, urine, saliva, feces, other, RNA, tissue (frozen)	100,000–1,000,000
Nl	CDDSS Knowledge base			
	**Collection types:** Cohort, disease specific, hospital			
	**Juridical person:** No information			
	Clinical Diagnostic Decision Support System on Anemia	Cohort, disease specific, hospital	Pathogen, plasma, whole blood	100–1,000
It	Cell line and DNA Biobank from patients affected by Genetic Diseases			
	**Collection types:** Case-Control, disease specific			
	**Juridical person:** Istituto Giannina Gaslini			
	Collection all Samples	Case-control, disease specific	Cell lines, DNA, other, plasma, serum, urine, RNA, whole blood	12,430
Nl	Parelsnoer			
	**Collection types:** Disease specific			
	**Juridical person:** No information			
	Parel Inflammatory bowel disease	Disease specific	DNA, feces, serum, tissue (frozen), tissue (paraffin preserved)	1,000–10,000
B	University Biobank Limburg			
	**Collection types:** Disease specific			
	**Juridical person:** University Hasselt/Jessa Hospital			
	University Biobank Limburg	Disease specific	DNA, other, plasma, RNA, serum, tissue (paraffin preserved), urine, whole blood, tissue (frozen), feces	14,431

## Lessons Learned—SWOT Analysis

SWOT analysis (syn. SWOT matrix) is a strategic planning tool used to help a person or organization identify strengths, weaknesses, opportunities, and threats related to business competition or project planning^4^. It is intended to identify the internal and external factors that are favorable and unfavorable to achieving those objectives.

The strengths of a biobank are obvious and numerous (Table 2). They are related to the number and especially the quality of the samples of HBM and specific features, associated data and procedures for access to samples. Quality is based on and identified as adherence to standard quality principles and procedures, certification, accreditation … Numerous systems do exist (e.g., ISBER, OECD, WHO, …). The Organization for Standardization (ISO) has set in 2018 specific requirements for bioresources for research i.e., Biotechnology—Biobanking—General requirements for biobanking (ISO 20387). Specific features may relate to the clinical origin, detailed information on the pre-analytical procedures, … which will determine the uniqueness or rarity of the HBM. Opportunities relate directly or indirectly to scientific outcome and examples of threats are disasters and the lack of contingency plans. The most commonly recognized “weakness” for biobank is sustainability, the difficulty of covering the total cost of the initiative independently of the economic model adopted. The primary support for biobanks is nearly evenly divided among grant support, public (government), and private funding at nearly 30% each (27, 28). Both the CMI initiative and the Dutch IBD Biobank went through a difficult time when external (public/governmental) funding stopped. In times of tight economic realities in research the need to discuss with stakeholders and reappraise financial models for biobanking is mandatory. Especially since biobanking has finally attained recognition as a key infrastructure for scientific research and clinical care.

**Table 2:** Analysis of strengths, weaknesses, opportunities, and threats of a biobank.

**Strenghts**	**Weaknesses**
Large number of samples Better quality of samples Presence of associated data Presence of access procedures	Sustainability vs. Non-profit setting “Ownership” Public trust Social acceptability Difficulty in implementing longitudinal sampling strategies Communication/marketing
**Opportunities**	**Threats**
Higher scientific output Development of innovative projects, new clinical trials, new diagnostics tested Integration of data Increased service provision Scientific collaboration with different partners	Lack of contingency plans Integration of big data incl. imaging data Accreditation requirements may increase cost structures

## Conclusions

A biobank collects, stores, processes, and distributes HBM and related health data for use in both fundamental research and clinical studies. The biobanking field has changed greatly over the last three decades, in general and in particular as demonstrated in this case study, starting with a university-based collection developed for the needs of particular project e.g., the VLECC—biobank. It then gradually evolved to an initiative supporting different projects, generating multicentric collaboration and an exponential increase in scientific output both in volume and in quality (impact factor and citation index). As described in this paper on IBD, biobank research does provide novel insights into amongst others the genetic component of disease, ultimately leading to a more personalized approach to healthcare.

As described in this article, long-term sustainability of biobanks remains a major concern. Literature review demonstrates that total cost-recovery strategies are not the best approach to reach and maintain sustainability. Biobanks will always require support by long-term investment and commitment, preferentially from public and governmental sources.

## Data Availability Statement

All datasets generated for this study are included in the article/supplementary material.

## Author Contributions

The authors as well as the acknowledged participants participated in the biobanking project and the realization of initiative. IC, LL, and NE wrote the article.

### Conflict of Interest

The authors declare that the research was conducted in the absence of any commercial or financial relationships that could be construed as a potential conflict of interest.

## Footnotes

^1^NIDDK. (2014). Archived from the original on 28 July 2016.

^2^White Paper of FlandersBio 2006 www.flanders.bio.

^3^Policy memorandum, Science and Innovation, Policy Priorities 2011–2012, Minister Lieten, 26 October 2011.

^4^SWOT Analysis: Discover New Opportunities, Manage and Eliminate Threats. www.mindtools.com.
